# Kidney Transplantation in Children Weighing Less than 15 kg: A 35-Year Single-Center Experience

**DOI:** 10.3390/jcm14144905

**Published:** 2025-07-10

**Authors:** Elisa Benetti, Nicola Bertazza Partigiani, Marco Moi, Maria Sangermano, Francesco Fascetti Leon, Luisa Meneghini, Marco Daverio, Federica De Corti

**Affiliations:** 1 Pediatric Nephrology, Department of Women’s and Children’s Health, Padua University Hospital, 35128 Padua, Italy; 2 Laboratory of Immunopathology and Molecular Biology of the Kidney, Institute of Pediatric Research “Città della Speranza”, Department of Women’s and Children’s Health, Padua University Hospital, 35128 Padua, Italy; 3 Pediatric Renal Transplant Center, Department of Women’s and Children’s Health, Padua University Hospital, 35128 Padua, Italy; 4 Pediatric Surgery, Department of Women’s and Children’s Health, Padua University Hospital, 35128 Padua, Italy; 5 Pediatric Anesthesiology, Department of Women’s and Children’s Health, Padua University Hospital, 35128 Padua, Italy; 6 Pediatric Intensive Care Unit, Department of Women’s and Children’s Health, Padua University Hospital, 35128 Padua, Italy

**Keywords:** children, kidney transplantation, long-term outcomes

## Abstract

**Background**: Kidney transplantation is the treatment of choice for pediatric patients with end-stage kidney disease. However, transplantation in children weighing < 15 kg remains challenging due to limited donor availability and higher surgical and medical risks. We report our 35-year single-center experience in this population, focusing on perioperative and long-term outcomes. **Methods**: We retrospectively analyzed kidney transplants performed from 1987 to 2023 in children weighing < 15 kg. Data on demographics, donor type, complications, immunosuppression, and outcomes at 2, 5, and 10 years (including survival, graft function, rejection, infections, and urological issues) were collected. Outcomes were compared between deceased and living donors and between recipients weighing < 10 kg and ≥10 kg. **Results**: Ninety-six transplants were included (mean age 3.3 years; mean weight 11.1 kg), 80 from deceased and 16 from living donors. Most patients (69.8%) had been treated with peritoneal dialysis. Median follow-up was 120 months. Patient survival was 95.8%; graft survival was 78.1%. Eight grafts (8.3%) were lost to renal vein thrombosis, all in deceased-donor recipients (*p* = 0.60). Preserved renal function (eGFR > 60 mL/min/1.73 m^2^) declined from 80.4% at 2 years to 66.0% at 5 years and 18.0% at 10 years. Graft survival at 10 years was significantly lower in children < 10 kg vs. ≥10 kg (49.6% vs. 80.3%, *p* = 0.003). CAKUT was associated with higher urological complication rates (*p* = 0.017). No significant differences emerged between living and deceased donor groups. **Conclusions**: Transplantation in children < 15 kg is feasible with good outcomes, but those <10 kg present lower graft survival at 10 years. Multidisciplinary assessment and center experience are key to optimizing results.

## 1. Introduction

Chronic kidney disease (CKD) in children is associated with increased mortality and contributes to significant long-term morbidity, including growth problems, cardiac dysfunction, neurodevelopmental delay, and reduced quality of life. While advancements in medical management and renal replacement therapy for pediatric patients with CKD have improved overall survival, renal transplantation remains the best approach for ensuring growth, neurocognitive and social development, and significantly improving the quality of life for patients and their caregivers [1]. Transplantation is also a more cost-effective therapy for the healthcare system. The only absolute contraindications for renal transplantation in children are uncontrolled malignancies and active infections. Therefore, the majority of experts recommend transplantation as soon as possible [2]. This is particularly true for small children, typically those weighing less than 15 kg, who have a high risk of mortality if maintained on dialysis [3].

Although advancements in surgical techniques and medical management have significantly reduced mortality and graft loss over time, renal transplantation in the smallest patients remains uncommon, mainly due to the limited availability of appropriately sized deceased donor allografts and the hesitancy regarding the use of small (<15 kg) or young (<5 years) donors [4]. To address these challenges, adult single kidneys have been increasingly used in small children to improve access to transplantation [3,5].

Despite the advantages of early transplantation, low-weight (<15 kg) recipients are considered a surgical, anesthesiological, and medical challenge. Potential surgical complications—arising from smaller vascular anastomosis, congenital vascular anomalies, and disparities in vessel or body size when adult donor organs are used—can lead to serious morbidity and graft loss [4]. Additionally, the management of fluids, antithrombotic therapy, and immunosuppressive treatment immediately after surgery, as well as post-transplant short- and long-term follow-up, requires a highly experienced multidisciplinary approach.

Nevertheless, numerous reports in the literature suggest that with proper pre-, intra-, and post-operative management, excellent outcomes can be achieved even in infants, outweighing the traditionally perceived risks of kidney transplantation in this group of patients [4].

In this paper, we report our experience with renal transplantation in children weighing < 15 kg, providing an overview of the challenges in surgical management and medical short- and long-term follow-up.

## 2. Materials and Methods

### 2.1. Study Design and Population

In our department, pediatric nephrology activities began in 1971, followed by the initiation of dialysis in 1973. The first kidney transplantation from a deceased donor was performed in 1987, and the first from a living donor in 1991.

This retrospective study included all pediatric patients weighing less than 15 kg who underwent kidney transplantation between April 1987 and January 2023. All clinical data were obtained from the medical records, and all patients’ legal guardians gave their written consent for the collection of these data.

Recorded parameters included demographic data, primary renal disease, pre-operative status (pre-emptive transplantation vs. renal replacement therapy), donor characteristics, surgical complications, and long-term outcomes at 2-, 5-, and 10-years post-transplantation. Outcome measures included patient and graft survival, incidence and causes of graft loss, renal function (evaluated by estimated Glomerular Filtration Rate (eGFR)), occurrence of rejection episodes (cell- or antibody-mediated), incidence of viral infectious complications (Epstein–Barr virus, BK virus, cytomegalovirus, adenovirus, and parvovirus B19), and urological complications (urinary tract infections, urinary dilatation, or obstruction).

### 2.2. Pre- and Post-Operative Care

A comprehensive preoperative evaluation, encompassing nephrological, pediatric, surgical, urological, anesthesiologic, nutritional, nursing, social, and psychological aspects, was carried out in all children. According to our internal protocol, many healthcare professionals are involved in the process, and the final assessment report is shared in a multidisciplinary meeting, during which critical issues are discussed to determine the best transplant strategy. Particular attention is given to conditions that may impact transplant outcomes, including surgical factors (such as patency of the neck and abdominal vessels and previous surgical history), urological aspects (particularly urinary tract dysfunctions), infectious status (including vaccination history), immunological status, hypercoagulability risk, cardiac function, comorbidities, nutritional status, overall therapy compliance, and family socioeconomic and psychological factors.

All children were admitted to the Pediatric Intensive Care Unit (PICU) for post-surgical observation. According to our practice, extubation occurred in the operating room or shortly after admission to the PICU. Recipients underwent 12–24 h of intensive observation before being transferred to the Pediatric Nephrology Unit.

Small children who underwent living-donor transplantation received aggressive hydration and inotropic support if necessary, as the adult-sized graft they received was estimated to absorb up to 50% of the small recipient’s blood volume per minute. Central venous pressure was closely monitored during the first hours after implantation and maintained at 8–15 mmHg after revascularization. Doppler ultrasound was performed daily after surgery for vascular complication surveillance until complete renal function recovery.

Immunosuppression was induced with Basiliximab and methylprednisolone, followed by maintenance therapy with prednisone, tacrolimus (or cyclosporine if tacrolimus was not well tolerated), and mycophenolate mofetil (or mTOR inhibitors). Antithymocyte globulins (ATG) were used in children undergoing a second transplant. Before 1999, immunosuppression was induced with methylprednisolone and maintained with cyclosporine, azathioprine, and prednisone. Due to the limited number of patients treated with azathioprine (*n* = 4), no comparative statistical analysis between azathioprine and mycophenolate mofetil regimens was performed.

### 2.3. Surgical Technique

According to our institutional kidney transplantation protocol, optical loupes with 2.5–4X magnification and microsurgical instruments were used. An extraperitoneal approach was employed for all patients. To ensure optimal visualization of the retroperitoneum and bladder, even in those with prior surgical history, a “hockey stick” incision was performed.

All grafts were implanted in the iliac fossa through an extraperitoneal access, carefully dissecting the peritoneum—which needed to remain intact—from the lateral abdominal wall and exposing the iliopsoas muscle and iliac vessels. The right side was preferred as the primary implantation site. However, in cases of severe previous urinary tract infections leading to inflammatory adhesions, the left iliac fossa was considered as an alternative.

To achieve optimal exposure of the vessels, one Collin’s and two Doyen’s abdominal retractors were used to medialize the peritoneum. This setup also facilitated adequate retraction of the liver, particularly in patients with hepatomegaly. The renal vein and artery were anastomosed in an end-to-side configuration, connecting them to the iliac vessels or, in low-weight patients, to the vena cava and aorta.

The ureterovesical anastomosis was performed using an extravesical approach following the Lich-Gregoir technique. A trans-anastomotic external stent was inserted into the renal pelvis in all patients to maintain anastomotic patency and to monitor urinary output from the transplanted kidney, especially in cases where residual diuresis from the native kidneys was present.

Routine protocol biopsies were conducted at 6-, 12-, and 24-months post-transplant, in addition to any biopsies performed as clinically indicated.

All small recipients were classified as having an intermediate risk for thrombotic complications due to the size mismatch (a donor-to-recipient body weight ratio greater than 1:4) and received unfractionated heparin via continuous infusion (starting dose: 5–10 IU/kg/h) immediately after implantation, continuing until the surgical drains were removed. Those with additional risk factors—including pathological preoperative coagulation screening, thrombotic disorders, previous thrombosis, donor kidneys with multiple vessels, or an intimal lesion of the allograft renal artery—continued antithrombotic prophylaxis with acetylsalicylic acid for up to six months [6].

### 2.4. Statistical Analysis

Continuous variables were expressed as the mean and standard deviation (SD) or as the median and interquartile range (IQR), depending on the distribution of the variable under study. Student’s *t*-test or the Mann-Whitney U-test was used for continuous variables, while Fisher’s exact test was employed for categorical variables, as appropriate. Kaplan–Meier survival curves were generated for patient and graft survival, and differences were evaluated with the log-rank test. A *p*-Value of less than 0.05 was considered statistically significant. Comparative analyses were performed between recipients of living-related and deceased donor kidneys, as well as between patients with a transplant weight of less than 10 kg and those weighing more than 10 kg. Finally, we used Cox proportional-hazards regression models in Jamovi 2.4 (The Jamovi Project) to quantify the independent effect of clinical and demographic covariates on time-to-event outcomes. For each endpoint (graft loss and decline of renal function), covariates with log-rank *p*  <  0.10 in univariate analyses were entered into multivariable Cox models. Due to the very low number of deaths observed, a multivariable Cox model for overall mortality could not be reliably estimated. Hazard ratios (HR) and 95% confidence intervals (CI) were reported, with two-sided *p*  <  0.05 considered statistically significant.

## 3. Results

### 3.1. Population Characteristics

Between April 1987 and January 2023, a total of 558 pediatric transplants were performed at our Centre, 96 (17.2%) of which were in children weighing <15 kg. The mean age was 3 years and 4 months (standard deviation (SD): ±1 year 5 months), with a mean weight of 11.1 kg (SD: ±2.4 kg). Transplantations from deceased donors were 80 (83.3%), and living-related transplants were 16 (16.6%). The causes of CKD in the children who underwent living-related transplantation were: Congenital Anomalies of the Kidney and the Urinary Tract (CAKUT) (64.6%), Congenital Nephrotic Syndrome (CNS) (15.6%), Autosomal Recessive Polycystic Kidney Disease (ARPKD) (8.3%), perinatal asphyxia (4.2%), and other kidney diseases (7.3%). Most transplants were not pre-emptive, and peritoneal dialysis was the most frequently used replacement therapy: 9.4% of children underwent pre-emptive transplantation, 69.8% received peritoneal dialysis, 10.4% hemodialysis, and the remaining 10.4% a combination of peritoneal dialysis and hemodialysis. The median donor/recipient (D/R) weight ratio was 3.9 (SD: ±1.8). Population characteristics divided according to the type of transplantation are presented in Table 1. The two populations differed significantly in the D/R weight ratio, as recipients from living donors had a ratio of 5.3 (SD: ±1.1), while those from deceased donors had a ratio of 3.0 (SD: ±1.7) (*p* < 0.001), and in HLA mismatch number, which was higher in deceased donors (4 vs. 3, *p* = 0.002).

### 3.2. Kidney Transplantation Outcome

The median follow-up duration in our cohort was 120 months (interquartile range (IQR): 72–120 months), with an overall survival rate of 95.8%. All patients were alive one month after kidney transplantation. Four patients subsequently died due to sepsis, intracranial hemorrhage, cardiac arrest secondary to acute bronchospasm, and bladder tumor. A total of 21 (21.9%) patients experienced graft loss during the follow-up period: 8 children—all in the deceased donor group (*p* = 0.60)—lost the graft due to renal vein thrombosis, while chronic humoral rejection accounted for 60% of causes in the 13 remaining cases. Eight patients underwent a second kidney transplant, and, to date, none of them have experienced a second graft loss. The long-term outcomes of living and deceased donor kidney transplantation are separately presented in Table 2, Table 3 and Table 4. At the 2-year follow-up, 80.4% of patients exhibited normal or only a mild reduction in kidney function (eGFR > 60 mL/min/1.73 m^2^), while 9.8% had moderate impairment (eGFR 30–60 mL/min/1.73 m^2^) and 9.8% had eGFR < 30 mL/min/1.73 m^2^. At 5 years, kidney function was slightly impaired, with eGFR > 60 mL/min/1.73 m^2^ in 66.0% of patients, eGFR 30–60 mL/min/1.73 m^2^ in 26.7% of children, and eGFR < 30 mL/min/1.73 m^2^ in 8.0% of recipients. After 10 years, 18% of patients exhibited normal function, while 60% showed a mild-to-moderate reduction in eGFR (30–90 mL/min/1.73 m^2^), and 22% a severe impairment (eGFR < 30 mL/min/1.73 m^2^).

Viral infections complicated 38.5% of patients within the first 2 years, with Epstein–Barr virus (EBV) being the most prevalent (18.7%). The incidence of viral infections increased to 42.1% after 5 years, primarily due to EBV (27.6%), with isolated cases of BK virus (BKV), cytomegalovirus (CMV), and Parvovirus B19 (PVB19). By the 10-year mark, viral infections decreased to 28%, with 20% represented by chronic EBV infection and one isolated case of BKV and two of PVB19.

Urological complications—including urinary tract infections, dilatation, or obstruction—occurred in 23% of patients within the first 2 years and declined over time (21.3% at 5 years and 14% at 10 years).

Considering the rejection episodes, 46.2% of patients experienced some form of rejection within the first 2 years post-transplant, with cellular rejection being the most prevalent (20.4%). This proportion slightly decreased after 5 years, with 19.7% of patients experiencing rejection, primarily antibody-mediated (11.8%), and after 10 years, only 12.0% of patients experienced rejection, mainly antibody-mediated (10.0%). Chronic histological lesions (interstitial fibrosis and tubular atrophy—IF/TA) were detected in only 2% of patients at 2 years but increased markedly over time, reaching 22% at 5 years and 40% at 10 years.

When comparing the living and deceased donor populations, a difference was observed at the 5-year point in the number of rejection episodes, which were more frequent in the living donor group (50% vs. 12.9%, *p* = 0.055). Furthermore, there was a higher proportion of IF/TA lesions at the same time point in the population of children who received the graft from a deceased donor (27.4% vs. 0%, *p* = 0.03). No other differences were observed (Table 2).

### 3.3. Survival Curves and Long-Term Clinical Outcomes

**Figure 1:**
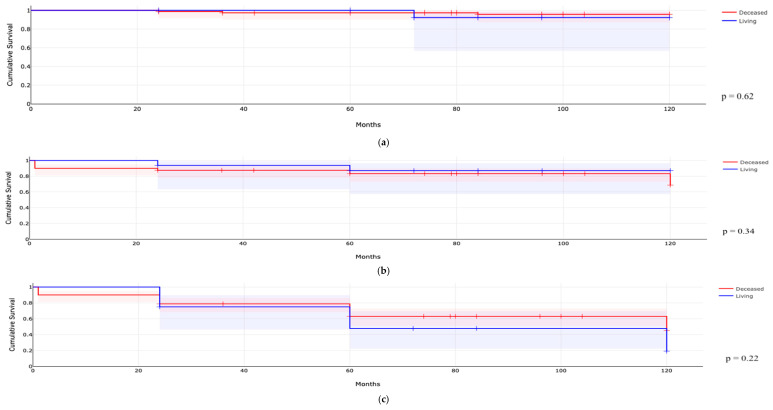
Difference in mortality ((**a**), **up**), graft survival ((**b**), **medium**), and eGFR < 60 mL/min/1.73 m^2^ ((**c**), **down**) in children receiving kidney transplantation from deceased or living donors.

**Figure 2:**
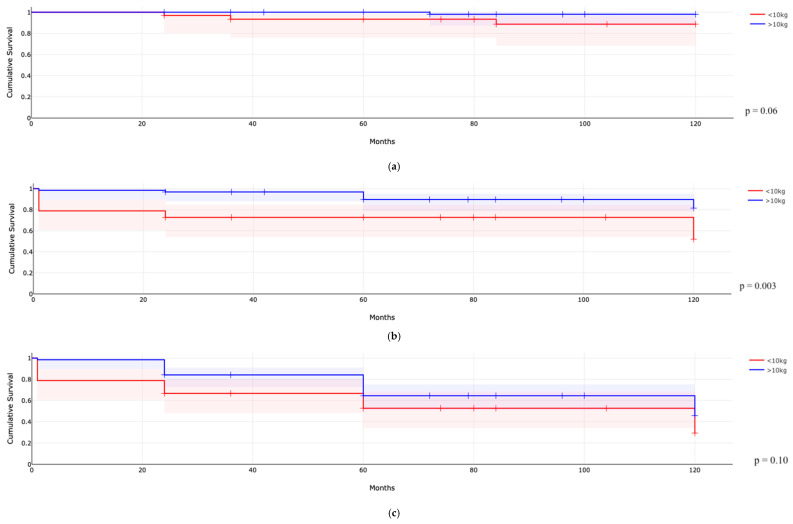
Difference in mortality ((**a**), **up**), graft survival ((**b**), **medium**), and eGFR < 60 mL/min/1.73 m^2^ ((**c**), **down**) in kidney recipient children weighted < or >10 kg.

**Figure 3:**
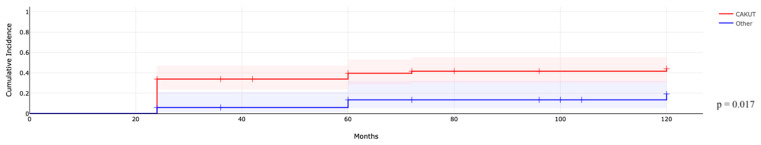
Incidence of urological complications in patients affected by CAKUT compared to other diagnoses, *p* = 0.017.

A comparison between children transplanted from living and deceased donors did not reveal significant differences in terms of mortality (Figure 1a, *p* = 0.62), graft failure (Figure 1b, *p* = 0.34), or the incidence of eGFR < 60 mL/min/1.73 m^2^ (Figure 1c, *p* = 0.22), as shown in Figure 1. Furthermore, these populations did not differ in other outcomes such as infections, rejection, IF/TA, or urological complications. Among patients with a transplant weight of less than 10 kg, reduced graft survival over time was observed compared to those weighing more than 10 kg (Figure 2b, <10 kg 49.6%, >10 kg 80.3% at 10 years, *p* = 0.003), and there was also a trend toward higher mortality (Figure 2a, <10 kg 11.8%, >10 kg 2.4% at 10 years, *p* = 0.06), while no significant difference was found in the incidence of eGFR < 60 mL/min/1.73 m^2^ (Figure 2c, *p* = 0.10). In multivariable Cox regression analyses reported in Table 5, transplant weight < 10 kg emerged as a strong independent predictor of graft failure (HR 3.38, 95% CI 1.42–8.08; *p* = 0.006), while postoperative urological complications were associated with a lower risk of graft loss (HR 0.17, 95% CI 0.04–0.72; *p* = 0.016). For a decline to eGFR  <  60 mL/min/1.73 m^2^, both weight < 10 kg (HR 2.41, 95% CI 1.16–4.99; *p* = 0.018) and biopsy-proven IF/TA (HR 2.54, 95% CI 1.29–5.02; *p* = 0.007) were independent risk factors. A Cox model for overall mortality could not be reliably estimated due to the very low number of deaths observed. Finally, patients with CAKUT experienced a higher incidence of urological complications compared to those with other diagnoses (*p* = 0.017), as reported in Figure 3. Finally, a survival analysis was conducted among patients weighing over 10 kg, differentiating between living and deceased donors. While no significant differences were observed in terms of patient or graft survival (*p* = 0.72 and *p* = 0.51, respectively), the incidence of eGFR < 60 ml/min/1.73 m^2^ showed a trend approaching statistical significance, being higher among recipients from living donors compared to deceased donors (79.2% vs. 45.7% after 10 years of follow-up, respectively; *p* = 0.07).

## 4. Discussion

This study analyzed kidney transplantation outcomes in children under 15 kg, highlighting critical factors for optimizing the clinical and surgical management of this particularly vulnerable population.

Overall, our data show excellent long-term outcomes, with no statistically significant differences between living- and deceased-donor transplantation, confirming the encouraging results previously reported in the literature [7,8,9,10,11,12,13,14]. Indeed, patient and graft survival rates at 2, 5, and 10 years for both living- and deceased-donor recipients were overall better or comparable to those reported in previous studies involving similar patient populations [3,4,13,14].

However, kidney transplantation in small children still raises several concerns, and many transplant centers prefer to operate only on children weighing over 15 kg. One of the main reasons is the risk of surgical complications, the most feared and harmful being graft thrombosis [15]. In our cohort, graft venous thrombosis occurred in 8.3% of patients, all of whom had received a deceased donor graft, consistent with previously reported NAPRTCS rates of 12% in failed index transplants and 14% in repeat transplants [13,14].

Given the importance of preventing complications, our smallest patients undergo extensive preoperative assessment, meticulous perioperative management, and routine infusion of unfractionated heparin. In our series, the median donor-to-recipient body weight ratio (D/R) was 3.9, but recipients of deceased donor grafts had a significantly lower D/R ratio (3.0 ± 1.7 vs. 5.3 ± 1.1, *p* < 0.001), which may partly explain the increased incidence of thrombosis in this group. At our Centre, a D/R ratio of 1.5–4 is considered optimal for both living- and deceased-donor transplantation. However, due to age- or disease-related growth retardation, maintaining this ratio can make it difficult to find a suitably matched organ for children under 15 kg. The availability of appropriately sized donor allografts is indeed limited, and the waiting time for small children is significantly longer than for older ones, even though organs from pediatric deceased donors are prioritized for children in our Country.

To improve access to transplantation, adult-sized kidneys have increasingly been used in small recipients [4,5], most often from living donors such as parents or close relatives (e.g., grandparents). Living-donor transplantation allows for earlier intervention, potentially avoiding CKD- and dialysis-related complications and supporting growth and development [16]. In our cohort, living-related transplantation in children under 15 kg was performed in a small percentage of patients (2.8%), mostly after the initiation of dialysis. Previous studies reported a wide range of living-donor transplants in infants, from 2% to as high as 75% of all pediatric kidney transplants [17,18,19]. In our study, the low number and non-preemptive timing of living-donor transplants were mainly due to surgical concerns—namely, excessively high D/R ratios (exceeding 5:1–6:1).

Although the use of adult-sized kidneys has reduced vascular complications compared to pediatric donor kidneys [18], and no vascular complications were observed in our living-donor recipients, our concern in infants receiving adult-sized kidneys is their inability to sustain the required renal blood flow. This not only increases the risk of vascular complications but may also contribute to delayed graft function. In line with existing literature, our internal protocol includes maintaining high central venous pressure through fluid infusions and administering inotropic agents when clinically indicated. Several studies have emphasized the importance of aggressive fluid administration and careful perioperative hemodynamic management, particularly during unclamping and reperfusion, when the adult-sized kidney may sequester a large proportion of the infant’s circulating blood volume [19].

Previous studies raised concerns about graft function in small children receiving adult versus pediatric grafts. While one-year graft survival tends to be poorer when both donor and recipient are very small—often due to early vascular or surgical complications—adult donor kidneys may experience downregulation to adjust to the recipient’s small body size, limiting their ability to increase absolute eGFR as the child grows [20,21]. Conversely, pediatric donor kidneys appear to better adapt their eGFR as the recipient grows [8,10]. Our findings support this hypothesis: in recipients weighing over 10 kg, living-donor grafts showed a trend toward lower eGFR at 10 years compared with deceased-donor grafts. Larger children are more likely to receive pediatric donor kidneys, particularly those with the “ideal” D/R weight ratio of 1.5–4 and a better capacity to increase absolute GFR during growth.

A particularly notable finding in our cohort is that children weighing less than 10 kg at transplant had significantly worse long-term outcomes compared to those over 10 kg, including lower patient and graft survival rates and worse eGFR at 2, 5, and 10 years. Previous studies have also analyzed outcomes in recipients under 15 or 10 kg. In a case series of 40 children weighing less than 11 kg, Becker et al. reported patient survival rates comparable to ours (93% at 1 year, 90% at 5 and 10 years), but better graft survival at 1 year (93%) and at 10 years (66%), with a thrombosis rate of 2% [9]. Another study of 63 recipients under 20 kg reported similar overall outcomes, but when stratified by weight (<11 kg vs. >11 kg), no significant differences were found, differently from our data [3]. In that study, thrombosis occurred in approximately 3% of patients, although it was not specified whether the cases involved the subgroup of the smallest children. A recent ESPN/ERA-EDTA Registry report on 100 children transplanted under 10 kg found similar 5-year patient and graft survival compared to children over 10 kg, though 1-year graft survival was significantly lower in the <10 kg group (90% vs. 95%) [21]. This suggests that the first year is the most vulnerable period, primarily due to vascular complications, whereas long-term outcomes may equalize if the graft survives the first year. Interestingly, the same study found low early graft loss in both groups when analyzing data from five countries responsible for over 80% of the transplants, likely due to the high expertise of those centers [21]. In our cohort, recipients under 10 kg had a 20% early graft loss rate due to thrombosis, compared to 2% in those over 10 kg, confirming the impact of vascular complications on short-term outcomes. Furthermore, the trend toward higher mortality, significantly increased long-term graft loss (HR 3.38, *p* = 0.006), and worse eGFR (HR 2.41, *p* = 0.018) in the smallest children suggests that additional factors influence long-term outcomes. These findings support the need for a thorough multidisciplinary evaluation of transplant eligibility in very small children, accounting for overall morbidity, mortality risk, and factors contributing to frailty of this particular subgroup of children, even in highly experienced transplant centres.

Post-transplant complications—including viral infections, urological complications, and rejection episodes—remain important follow-up concerns. Infections are a major cause of morbidity, with viruses playing a key role both directly and through their impact on graft survival. In our cohort, viral infections occurred in nearly 40% of patients within the first two years, with EBV being the most common (18.7%). The incidence rose to 42% at 5 years, still mostly due to EBV (27.6%), while BKV, CMV, and PVB19 remained uncommon. At 10 years, viral infections decreased to 28%, with chronic EBV (20%) remaining the most prevalent, alongside isolated cases of BKV and PVB19.

Infectious complications are categorized by time of onset: early (0–30 days), intermediate (31–180 days), and late (>180 days) [22,23]. Early infections are mostly bacterial and often related to surgical or nosocomial exposure. Intermediate infections typically arise from latent viruses transmitted via the graft or reactivated in previously exposed recipients (e.g., EBV, CMV), and late infections often follow cessation of antiviral prophylaxis. Returning to home and school increases exposure to community-acquired infections. Subclinical EBV-DNAemia has been reported in up to 40% of patients in the first post-transplant year, with symptomatic infections often linked to primary donor-derived infections. Risk factors include donor-recipient (D/R) serostatus and young recipient age; many young recipients are seronegative and receive a seropositive organ (D+/R−) [23]. While living donors—being adults—are more likely to be seropositive, we observed only a slightly higher, non-significant frequency of EBV in living-donor recipients.

CMV incidence was low in our cohort. Without prophylaxis, CMV occurs in 40–100% of transplant recipients; current preventive strategies have reduced this to 14–37%, with the highest risk in the first 100 days [24]. Since risk is primarily determined by D/R serostatus (highest: D+/R−), we prescribe valganciclovir in such mismatches, likely explaining the low rate of late-onset CMV in our patients. Similarly, PVB19 and BKV infections were rare and occurred late. Both viruses are typically acquired during childhood; about 80–90% of adults have been exposed. Risk factors include young age and low virus-specific antibody titres. Asymptomatic BKV viremia is detected in 10–20% of patients during the first year, but BKV-associated nephropathy occurs in only 1–10% of cases [23]. In our previous study, PVB19 positivity was seen in fewer than 5% of children. Both viruses can be transmitted via the graft, particularly in young children, due to donor latency and recipient immunosuppression [25].

In summary, the high incidence of viral infections—particularly EBV—within two years post-transplant underscores the vulnerability of children under 2 years of age, as supported by other studies reporting higher infection rates in young children compared to adolescents due to seronegativity at transplant [26].

Interestingly, our data show consistently higher rejection rates in the living-donor group than in the deceased-donor group, with a statistically significant difference at 5 years (50.0% vs. 12.9%, *p* = 0.005). Although this trend did not reach significance at 2 and 10 years, rejection remained numerically higher in the living-donor group across all time points. This is somewhat unexpected, as living-donor transplantation is usually associated with better outcomes, including lower rejection rates. Since both groups received the same immunosuppressive protocol and clinical monitoring, management differences are unlikely to explain this. Notably, deceased donor recipients had more HLA mismatches than living-donor recipients (median 4 vs. 3, *p* = 0.002), making differences in immunologic compatibility an unlikely explanation. One possible factor may be differences in immunosuppressive drug exposure; however, we lacked complete data on blood drug levels, which limits our ability to fully explore this potential explanation for the higher rejection rate observed in the living-donor group. Some viruses, such as PVB19, have been implicated in humoral alloimmunity and antibody-mediated rejection [25], but we observed no significant differences in viral infection rates between donor groups. Another finding in our population is higher evidence of IF/TA in the deceased donor group than the living donor group at 5 years (27.4% vs. 0%, *p* = 0.03), despite similar renal function between the two groups. IF/TA is a multifactorial, progressive structural lesion that can advance even in the absence of apparent functional decline. Kidneys from deceased donors are more exposed to donor-, recipient-, and transplant-related factors (including variables not captured in our analysis, such as cold ischemia time and ischemia-reperfusion injury), which are known to activate inflammatory and fibrogenic pathways even without overt graft dysfunction. Furthermore, IF/TA represents a risk factor (HR 2.54, *p* = 0.007) to the decline of function (eGFR < 60 mL/min/1.73 m^2^), which is consistent with the observation that children with histological evidence of chronic lesions experience worse kidney function over time [27].

Another important finding concerns patients with CAKUT, who had a higher incidence of urological complications, though these decreased over time. CAKUT, along with BKV, posterior urethral valves, and prior ureteral surgery, has been identified as a risk factor for urological complications [28,29]. In a recent study by our group, both CAKUT and pre-existing lower urinary tract dysfunction (LUTD) were associated with urological complications. However, no significant age-related differences were observed [30]. Still, special attention is warranted for infants and young children, as LUTD may be harder to detect in those without sphincter control. The decreasing incidence of urological complications over time in our cohort supports the hypothesis that they may stem from anatomical factors related to small body size. A thorough pre-transplant urological assessment can help minimize these risks and guide patient-tailored interventions [31]. Another curious result is that urological complications seem to be protective for graft failure (HR 0.17); however, a possible explanation is represented by an intensified follow-up in patients with urological complications, which may improve their post-transplantation management [30].

A limitation of our study is the inability to assess other factors (such as comprehensive data about deceased donors, feeding difficulties, malnutrition, dialysis-related complications, comorbidities, and psychosocial issues) that may further hinder transplantation in small children and contribute to their worse outcomes. Taken together, these challenges can lead to disparities in transplant access. Selective criteria for deceased donor organs (e.g., HLA compatibility, ideal donor size, absence of vascular anomalies) and size mismatch limitations in living donation may significantly narrow the transplant window. Fewer transplants may also limit team expertise. Another limitation is the small sample size, particularly in the living-donor group, and the unavailability of follow-up data on growth and cardiac function in many of the patients. However, transplants in children under 15 kg remain rare, emphasizing the need for multicenter studies to generate more robust data.

In conclusion, our study confirms that kidney transplantation in children under 15 kg yields good medium- and long-term outcomes. However, children under 10 kg exhibit worse long-term mortality and graft survival. A comprehensive multidisciplinary assessment of transplant eligibility is therefore essential, given the unique challenges of this vulnerable population, even in high-expertise transplant centers.

## Figures and Tables

**Table 1:** Characteristics of Padua’s Children Hospital cohort of transplanted children between April 1987 and June 2022 with weight lower than 15 kg divided based on the kind of donor.

	Living	Deceased	*p* Value
Population	*n* = 16	*n* = 80	
Mean age	4 ± 1.6 years	3 ± 1.3 years	0.41
Weight	13.2 ± 1.7 kg	10.7 ± 2.4 kg	0.65
Donor/Recipient Weight Ratio	5.3 ± 1.1	3.0 ± 1.7	**>0.001**
HLA mismatch	3 (IQR 2–3)	4 (IQR 3–4)	**0.002**
Median follow up	84 (IQR 72–120) months	120 (IQR 80–120) months	0.18
Overall survival rate	15 (94.7%)	77 (96.3%)	0.75
Underlying disease			
CAKUT	10 (62.5%)	52 (65%)	Overall *p* = 0.32
Congenital Nephrotic Syndrome	3 (18.8%)	12 (15%)	
Neonatal asphyxia	2 (12.5%)	2 (2.5%)	
Autosomal recessive polycystic kidney	1 (6.2%)	7 (8.8%)	
Other diseases	0 (0%)	7 (8.7%)	
Kidney replacement treatment before transplantation			
Conservative	4 (25%)	5 (6.2%)	Overall *p* = 0.16
Peritoneal dialysis	9 (56.2%)	58 (72.5%)	
Hemodialysis	1 (6.3%)	9 (11.3%)	
Both peritoneal and hemodialysis	2 (12.5%)	8 (10.0%)	

**Table 2:** Follow up at 2 years separated by type of donor (living and deceased).

	Living	Deceased	*p* Value
2 years follow up	*n* = 16	*n* = 80	
Survival rate	16 (100%)	79 (99%)	1
Graft failure	1 (6.3%)	10 (12.7%)	0.68
Second transplantation	0 (0%)	2 (2.5%)	1
Kidney function			
eGFR > 60 mL/min/1.73 m^2^	12 (75.0%)	65 (81.8%)	Overall *p* = 0.39
eGFR 30–60 mL/min/1.73 m^2^	3 (18.8%)	6 (7.8%)	
eGFR < 30 mL/min/1.73 m^2^	1 (6.2%)	9 (10.4%)	
Viral infections	6 (37.5%)	32 (40.2%)	0.8
Urological complications	4 (25.0%)	19 (23.4%)	1
Rejection episodes	9 (56.2%)	32 (40.2%)	0.32
IF/TA	0 (0%)	2 (2.5%)	1

**Table 3:** Follow up at 5 years separated by type of donor (living and deceased).

	Living	Deceased	*p* Value
5 years follow up	*n* = 14	*n* = 68	
Survival rate	14 (100%)	67 (99%)	1
Graft failure	1 (7.1%)	9 (13.4%)	1
Second transplantation	0 (0%)	5 (7.5%)	0.52
Kidney function			
eGFR > 60 mL/min/1.73 m^2^	7 (50.0%)	42 (61.8%)	Overall *p* = 0.20
eGFR 30–60 mL/min/1.73 m^2^	6 (42.9%)	12 (18.1%)	
eGFR < 30 mL/min/1.73 m^2^	1 (7.1%)	4 (6.5%)	
Viral infections	8 (57.1%)	38 (6.4%)	1
Urological complications	2 (14.2%)	15 (22.6%)	0.62
Rejection episodes	7 (50.0%)	9 (12.9%)	**0.005**
IF/TA	0 (0%)	19 (27.4%)	**0.03**

**Table 4:** Follow up at 10 years separated by type of donor (living and deceased).

	Living	Deceased	*p* Value
10 years follow up	*n* = 8	*n* = 50	
Survival rate	7 (85.7%)	49 (98%)	0.23
Graft failure	0 (0%)	14 (28.0%)	0.17
Second transplantation	0 (0%)	6 (12.0%)	1
Kidney function			
eGFR > 60 mL/min/1.73 m^2^	2 (28.6%)	25 (51.2%)	Overall *p* = 0.18
eGFR 30–60 mL/min/1.73 m^2^	4 (57.1%)	13 (25.6%)	
eGFR < 30 mL/min/1.73 m^2^	1 (14.3%)	11 (23.3%)	
Viral infections	3 (42.8%)	13 (25.6%)	0.38
Urological complications	1 (14.3%)	8 (16.3%)	1
Rejection episodes	2 (28.6%)	4 (9.3%)	0.19
IF/TA	3 (42.8%)	19 (39.5%)	1

**Table 5:** Main results in univariate and multivariate Cox regression analysis.

Endpoint	Covariate	Univariable HR (95% CI)	*p*-Value	Multivariable HR (95% CI)	*p*-Value
Graft failure	Weight < 10 kg	3.21 (1.35–7.64)	0.003	3.38 (1.42–8.08)	0.006
	Urological complications	0.18 (0.04–0.76)	0.02	0.17 (0.04–0.72)	0.016
	Any viral infection	2.17 (0.90–5.25)	0.08	2.17 (0.90–5.26)	0.086
Decline of function (eGFR < 60 mL/min/1.73 m^2^)	Donor type: deceased vs. living	0.57 (0.29–1.12)	0.10	0.59 (0.18–1.03)	0.056
	Weight < 10 kg	1.69 (0.93–3.09)	0.09	2.41 (1.16–4.99)	0.018
	IF/TA	2.20 (1.21–4.00)	0.009	2.54 (1.29–5.02)	0.007

## Data Availability

The datasets are not publicly available due to privacy and ethical restrictions. However, they are available from the corresponding author on reasonable request and with permission from the Ethics Committee, if applicable.

## Abbreviations

The following abbreviations are used in this manuscript:

ARPKD	Autosomal Recessive Polycystic Kidney Disease
ATG	Antithymocyte Globulin
BKV	BK Virus
CAKUT	Congenital Anomalies of the Kidney and Urinary Tract
CKD	Chronic Kidney Disease
CMV	Cytomegalovirus
CNS	Congenital Nephrotic Syndrome
D/R	Donor/Recipient
EBV	Epstein–Barr Virus
eGFR	Estimated Glomerular Filtration Rate
HLA	Human Leukocyte Antigen
IF/TA	Interstitial Fibrosis and Tubular Atrophy
IQR	Interquartile Range
LUTD	Lower Urinary Tract Dysfunction
mTOR	Mammalian Target of Rapamycin
NAPRTCS	North American Pediatric Renal Trials and Collaborative Studies
PICU	Pediatric Intensive Care Unit
PVB19	Parvovirus B19
RRT	Renal Replacement Therapy
SD	Standard Deviation
